# Supplementary material for: Analysis of PPARγ Signaling Activity in Psoriasis
Source: Int J Mol Sci. 2021 Aug 10;22(16):8603. doi: 10.3390/ijms22168603 (PMC8395241; doi:10.3390/ijms22168603)
Supplement: Supplementary file 1 [file ijms-22-08603-s001.zip › Supplemental materials_Analysis of PPARg signaling activity in psoriasis/Pathway models/Models images and html files/Interleukin-17 and interleukin-22 signaling in psoriasis/44555.html]

IL22RA1


# Protein IL22RA1

|  |  |
| --- | --- |
| URN | urn:agi-llid:58985 |
| Total Entities | 0 |
| Connectivity | 444 |
| Name | IL22RA1 |
| Description | interleukin 22 receptor subunit alpha 1 |
| Notes | The protein encoded by this gene belongs to the class II cytokine receptor family, and has been shown to be a receptor for interleukin 22 (IL22). IL22 receptor is a protein complex that consists of this protein and interleukin 10 receptor, beta (IL10BR/CRFB4), a subunit also shared by the receptor complex for interleukin 10 (IL10). This gene and interleukin 28 receptor, alpha (IL28RA) form a cytokine receptor gene cluster in the chromosomal region 1p36. [provided by RefSeq, Jul 2008] |
| Primary Cell Localization | Plasma membrane |
| Class | Receptor |

---

|  |  |
| --- | --- |
| Pathway | AHR in Intestinal Cell Antimicrobial Barrier Maintenance |
|  | IL22 Induces Keratinocyte Proliferation in Psoriasis |
|  | Keratinocyte Activation in Psoriatic Arthritis |
|  | Elevated Receptors -> Expression Targets in Colon |
|  | STAT3 and NFkB Activate Inflammation-Induced Tumorigenesis |
|  | IL22R -> STAT3 Signaling |
|  | IL22 Expression Targets |
|  | 2\_Interleukin-17 and interleukin-22 signaling in psoriasis |
|  | 1\_Inflammatory mediators increase the production of hepcidin (HAMP) |
|  | Interleukin-17 and interleukin-22 signaling in psoriasis |

---

|  |  |
| --- | --- |
| MedScan ID | 58985 |

---

|  |  |
| --- | --- |
| LocusLink ID | 58985 |
|  | 230828 |
|  | 362629 |

---

|  |  |
| --- | --- |
| Alias | CRF2 IX |
|  | OTTMUSP00000010252 |
|  | interleukin IL22RA1 |
|  | IL-22R-alpha-1 |
|  | RP23-110F21.4 |
|  | IL22 receptors |
|  | IL22 Rs |
|  | interleukin IL22 receptor |
|  | interleukin IL22R1 |
|  | IL22 receptor, alpha I |
|  | IL-TIF receptors |
|  | IL22 receptor, alpha 1 |
|  | IL-22 receptor |
|  | interleukin IL22 receptor, alpha 1 |
|  | interleukin IL22 Rs |
|  | IL10-related T-cell derived inducible factor receptor |
|  | RIKEN cDNA 9130219A07 gene |
|  | interleukin 22 receptor, alpha I |
|  | IL22R1 |
|  | 9130219A07Rik |
|  | LOC279229 |
|  | IL-22 receptor subunit alpha-1 |
|  | interleukin IL22 R |
|  | RGD1559655 |
|  | IL-TIF receptor |
|  | IL22 receptor, alpha |
|  | interleukin IL22RA I |
|  | IL22RA1 |
|  | interleukin 22 receptors |
|  | CRF2-9 |
|  | interleukin IL22 receptor, alpha I |
|  | interleukin-22 receptor subunit alpha-1 |
|  | OTTHUMP00000003073 |
|  | interleukin IL22 receptor, alpha |
|  | interleukin 22 receptor, alpha 1 |
|  | IL22RA I |
|  | IL22 R |
|  | cytokine receptor class-II member 9 |
|  | interleukin 22 receptor |
|  | interleukin IL22 receptors |
|  | interleukin 22 receptor, alpha |
|  | zcytoR11 |
|  | cytokine receptor family 2 member 9 |
|  | ENSMUSG00000073746 |
|  | IL22R |
|  | IL-22RA1 |
|  | IL-22R |
|  | interleukin-22 receptor alpha chain |

---

|  |  |
| --- | --- |
| GO ID | 0004896 |
|  | 0004904 |
|  | 0042015 |
|  | 0019221 |
|  | 0050829 |
|  | 0016021 |
|  | 0005886 |
|  | 0016020 |

---

|  |  |
| --- | --- |
| KEGG ID | hsa:58985 |
|  | mmu:230828 |

---

|  |  |
| --- | --- |
| Organism | Homo sapiens {Organism urn:agi-taxid:9606} |
|  | Mus musculus {Organism urn:agi-taxid:10090} |
|  | Rattus norvegicus {Organism urn:agi-taxid:10116} |
|  | Homo sapiens |
|  | Mus musculus |
|  | Rattus norvegicus |

---

|  |  |
| --- | --- |
| Mouse chromosome position | 4 67.99 cM |

---

|  |  |
| --- | --- |
| OMIM ID | 605457 |

---

|  |  |
| --- | --- |
| Rat chromosome position | 5q36 |

---

|  |  |
| --- | --- |
| Hugo ID | 13700 |
|  | HGNC:13700 |

---

|  |  |
| --- | --- |
| Human chromosome position | 1p36.11 |

---

|  |  |
| --- | --- |
| Swiss-Prot Accession | Q8N6P7 |
|  | Q8N6P7.1 |
|  | Q3TS18 |
|  | Q80XZ4 |
|  | Q80XZ4.1 |
|  | D3ZIM3 |
|  | A8K839 |
|  | B2R9Y9 |
|  | Q9HB22 |
|  | B2RU51 |
|  | Q3URP9 |

---

|  |  |
| --- | --- |
| GenBank ID | NC\_000001 |
|  | XM\_011541882 |
|  | XP\_011540184 |
|  | NM\_021258 |
|  | NP\_067081 |
|  | AL590683 |
|  | AL591178 |
|  | CH471134 |
|  | EAW95111 |
|  | AF286095 |
|  | AAG22073 |
|  | AK226001 |
|  | AK292204 |
|  | BAF84893 |
|  | AK304454 |
|  | BAG65271 |
|  | AK308522 |
|  | AK313971 |
|  | BAG36686 |
|  | BC014096 |
|  | BC029273 |
|  | AAH29273 |
|  | Q8N6P7 |
|  | NC\_000070 |
|  | NM\_178257 |
|  | NP\_839988 |
|  | AL662911 |
|  | CH466552 |
|  | EDL29970 |
|  | CQ889915 |
|  | CAH61680 |
|  | CQ889977 |
|  | CAH61684 |
|  | CS425176 |
|  | CAL60110 |
|  | CS719569 |
|  | CAP40191 |
|  | HC888946 |
|  | CBM42928 |
|  | AK141289 |
|  | BAE24639 |
|  | AK162333 |
|  | BAE36858 |
|  | AY103454 |
|  | AAM52222 |
|  | BC140972 |
|  | AAI40973 |
|  | BC140973 |
|  | AAI40974 |
|  | Q80XZ4 |
|  | NC\_005104 |
|  | NM\_001191869 |
|  | NP\_001178798 |
|  | AC\_000073 |
|  | AABR07050165 |
|  | AABR07050166 |
|  | AABR07073177 |
|  | AAHX01040257 |
|  | AAHX01040258 |
|  | AC135901 |
|  | CH473968 |
|  | EDL80765 |
|  | EDL80766 |
|  | NC\_018912 |
|  | AMYH02000517 |
|  | JF432213 |
|  | ADZ15430 |
|  | AC\_000026 |
|  | AAHY01040791 |
|  | AAHY01040792 |
|  | AC\_000133 |
|  | ABBA01059898 |
|  | ABBA01059899 |

---

|  |  |
| --- | --- |
| Swiss-Prot ID | I22R1\_HUMAN |
|  | I22R1\_MOUSE |

---

|  |  |
| --- | --- |
| Cell Localization | Membrane |

---

|  |  |
| --- | --- |
| Ensembl ID | ENSG00000142677 |
|  | ENSP00000270800.1 |
|  | ENST00000270800.2 |
|  | ENSMUSG00000037157 |
|  | ENSMUSP00000099605.3 |
|  | ENSMUST00000102546.3 |
|  | ENSRNOG00000027976 |
|  | ENSRNOP00000037743.4 |
|  | ENSRNOT00000031512.6 |
|  | ENSP00000270800 |
|  | ENST00000270800 |
|  | ENSMUSP00000099605 |
|  | ENSMUST00000102546 |
|  | ENSRNOP00000037743 |
|  | ENSRNOT00000031512 |

---

|  |  |
| --- | --- |
| MGI ID | MGI:2663588 |

---

|  |  |
| --- | --- |
| RGD ID | 1559655 |

---

|  |  |
| --- | --- |
| Unigene ID | Hs.110915 |
|  | Mm.261647 |
|  | Rn.95852 |

---

|  |  |
| --- | --- |
| Homologene ID | 10943 |

---

|  |  |
| --- | --- |
| Shape | Stick-vertex |

---
